# Enhancing the differentiation of pulmonary lymphoma and fungal pneumonia in hematological patients using texture analysis in 3-T MRI

**DOI:** 10.1007/s00330-020-07137-5

**Published:** 2020-08-21

**Authors:** Damon Kim, Thomas Elgeti, Tobias Penzkofer, Ingo G. Steffen, Laura J. Jensen, Stefan Schwartz, Bernd Hamm, Sebastian N. Nagel

**Affiliations:** 1 Klinik für Radiologie, Charité – Universitätsmedizin Berlin, Campus Benjamin Franklin, Hindenburgdamm 30, 12203 Berlin, Germany; 2 Klinik für Nuklearmedizin, Charité – Universitätsmedizin Berlin, Campus Virchow-Klinikum, Augustenburger Platz 1, 13353 Berlin, Germany; 3 Berlin Institute of Health (BIH), Anna-Louisa-Karsch-Str. 2, 10178 Berlin, Germany; 4 Medizinische Klinik mit Schwerpunkt Hämatologie und Onkologie, Charité – Universitätsmedizin Berlin, Campus Benjamin Franklin, Hindenburgdamm 30, 12203 Berlin, Germany

**Keywords:** Magnetic resonance imaging, Lymphoma, Pulmonary fungal infections, Differential diagnosis

## Abstract

**Objectives:**

To evaluate texture analysis in nonenhanced 3-T MRI for differentiating pulmonary fungal infiltrates and lymphoma manifestations in hematological patients and to compare the diagnostic performance with that of signal intensity quotients (“nonenhanced imaging characterization quotients,” NICQs).

**Methods:**

MR scans were performed using a speed-optimized imaging protocol without an intravenous contrast medium including axial T2-weighted (T2w) single-shot fast spin-echo and T1-weighted (T1w) gradient-echo sequences. ROIs were drawn within the lesions to extract first-order statistics from original images using HeterogeneityCAD and PyRadiomics. NICQs were calculated using signal intensities of the lesions, muscle, and fat. The standard of reference was histology or clinical diagnosis in follow-up. Statistical testing included ROC analysis, clustered ROC analysis, and DeLong test. Intra- and interrater reliability was tested using intraclass correlation coefficients (ICC).

**Results:**

Thirty-three fungal infiltrates in 16 patients and 38 pulmonary lymphoma manifestations in 19 patients were included. Considering the leading lesion in each patient, diagnostic performance was excellent for T1w entropy (AUC 80.2%; *p* < 0.005) and slightly inferior for T2w energy (79.9%; *p* < 0.005), T1w uniformity (79.6%; *p* < 0.005), and T1w energy (77.0%; *p* < 0.01); the best AUC for NICQs was 72.0% for T2NICQmean (*p* < 0.05). Intra- and interrater reliability was good to excellent (ICC > 0.81) for these parameters except for moderate intrarater reliability of T1w energy (ICC = 0.64).

**Conclusions:**

T1w entropy, uniformity, and energy and T2w energy showed the best performances for differentiating pulmonary lymphoma and fungal pneumonia and outperformed NICQs. Results of the texture analysis should be checked for their intrinsic consistency to identify possible incongruities of single parameters.

**Key Points:**

*• Texture analysis in nonenhanced pulmonary MRI improves the differentiation of pulmonary lymphoma and fungal pneumonia compared with signal intensity quotients.*

*• T1w entropy, uniformity, and energy along with T2w energy show the best performances for differentiating pulmonary lymphoma from fungal pneumonia.*

*• The results of the texture analysis should be checked for their intrinsic consistency to identify possible incongruities of single parameters.*

## Introduction

The differential diagnosis of pulmonary lesions can be challenging, particularly when it becomes necessary to distinguish infections from manifestations of the underlying condition in hematological patients [1]. Morphological findings are usually considered unspecific, especially that of pulmonary lymphoma and invasive bronchopulmonary aspergillosis [2–4]. The identification of primary pulmonary lymphoma can be particularly challenging, as the incidence is very low, i.e., < 1% of all non-Hodgkin lymphomas and 0.5% of all primary pulmonary malignancies [5, 6]. In a retrospective study, 13 of 19 patients with pulmonary lymphoma manifestations were initially misdiagnosed as having pneumonia, lung cancer, or tuberculosis [7].

Histopathologic workup is usually required to establish the correct diagnosis, but transthoracic biopsy is often difficult and requires the patient to be able to cooperate [8]. In hematological patients, thrombocytopenia could further increase the risk of complications of invasive diagnostic procedures, and, if sedation or general anesthesia is required, patients are put at an additional risk [9]. Therefore, noninvasive tools to improve the differentiation of unclear pulmonary lesions are desirable. Due to its excellent soft tissue contrast, MRI is being investigated in this context, e.g., by using DWI [10–12] or DCE-MRI [13, 14]. A straightforward approach proposed by Nagel et al shows encouraging results for differentiating infectious and noninfectious pulmonary lesions using simple signal intensity quotients (referred to as “nonenhanced imaging characterization quotients,” NICQs) from 3-T MR images [15]. However, the best parameters did not exceed an AUC of 80%. Theoretically, the diagnostic performance of 3-T MRI may be further enhanced by using texture analysis [16]. For this purpose, freely available software exists, e.g., HeterogeneityCAD and PyRadiomics [17, 18].

The aim of the present study was to evaluate texture analysis of nonenhanced MR imaging at 3 T for differentiating fungal infiltrates and pulmonary lymphoma manifestations in hematological patients. The diagnostic performance should further be compared with that of NICQs.

## Materials and methods

### Patients

This monocentric prospective study was approved by the local ethics committee (EA4/017/14). All patients were consecutively included and gave written informed consent. Data on NICQs in this patient collective have been published recently [19].

**Fig. 1:**
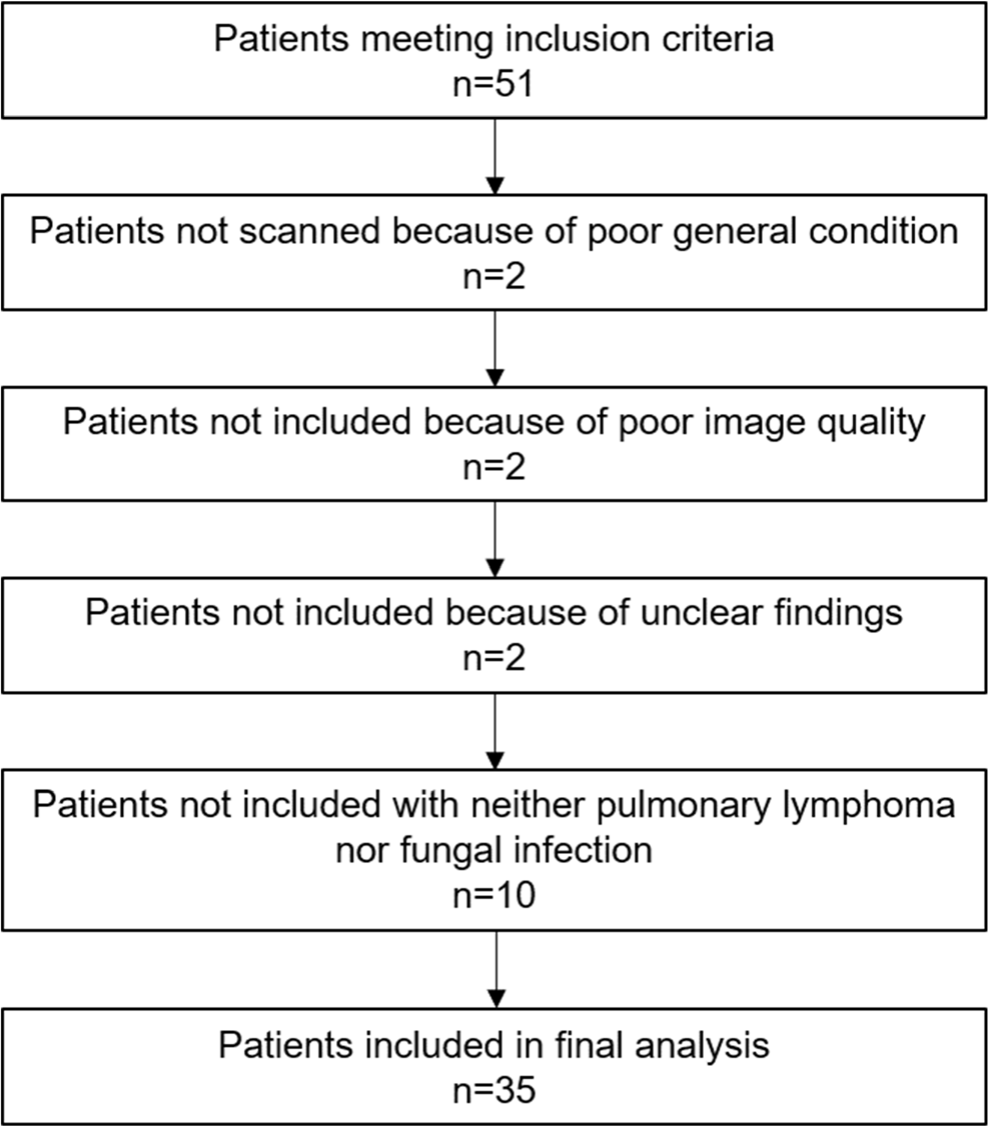
Flow chart displaying the inclusion and exclusion of patients in this study

The main inclusion criteria were an underlying hematological disease and the presence of at least one solid pulmonary lesion in a current, clinically indicated chest X-ray or CT scan; patients with contraindications to MRI were excluded. Pulmonary lymphoma manifestations had to be histopathologically proven or show unequivocal response to antineoplastic treatment during follow-up. Fungal infections had to be at least “probable” according to the European Organization for Research and Treatment of Cancer/Invasive Fungal Infections Cooperative Group and the National Institute of Allergy and Infectious Diseases Mycoses Study Group (EORTC/MSG) Consensus Group [20]. The final diagnosis was based on all available clinical data and established by a senior consultant oncologist (S.S.). All patients were included and scanned between April 2014 and July 2018. Sixteen patients of the initial collective of 51 patients were excluded: 2 patients with poor general condition not able to complete the examination, 2 patients due to poor image quality, 2 patients, in which the reliable attribution of the findings to lymphoma or fungal infection was ultimately not possible, and 10 patients with neither fungal infection nor lymphoma manifestation (Fig. 1).

### MRI technique

All MRI examinations were performed on a 3-T scanner (Magnetom Skyra, Siemens Healthineers). The patients were imaged in supine position using an MRI protocol derived from Biederer et al and Attenberger et al with a surface coil on the chest [21, 22].

Texture analysis was performed using imaging data acquired with a T2-weighted (T2w) single-shot fast spin-echo sequence (time of echo, 27 ms; time of repetition, 500 ms; refocusing flip angle, 160° after initial 90° excitation pulse; matrix size, 256 × 320; slice thickness, 5 mm) and a T1-weighted (T1w) gradient-recalled echo sequence (time of echo, 2.04 ms; time of repetition, 5.39 ms; flip angle, 9°; matrix size, 180 × 320; slice thickness, 3 mm), both in axial plane. A multi-breath-hold regimen was applied, aiming for a max breath-hold time of 8–10 s. An additional T2w single-shot fast spin-echo sequence in coronary plane was acquired for planning purposes, but not considered in the analysis. It has been shown that the protocol was suitable for immunocompromised patients [23].

### Image analysis

Image analysis was performed by two readers: a board-certified radiologist (S.N.) with more than 7 years of experience in cross-sectional imaging and, to evaluate interrater variability, by a radiology resident (D.K.) with more than 3 years of experience in MRI. For determination of intrarater variability, the less experienced reader repeated the image analysis in 9 cases of fungal infection and 9 cases of lymphoma manifestations.

**Fig. 2:**
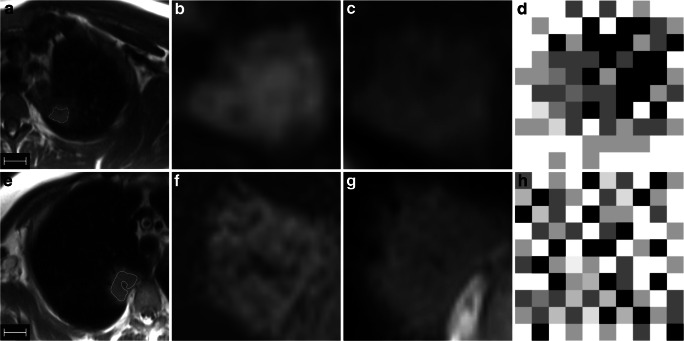
Upper row: 27-year-old male patient with acute myeloid leukemia and focal *Aspergillus* infiltrate in the left upper lobe; **a** T2w overview, **b** T1w, and **c** T2w with zoom on the lesion. Note the surrounding halo in (**a**), which was ignored when drawing the ROI. Scale indicates 2 cm. Lower row: 58-year-old female patient with gastric lymphoma originating from mucosa-associated lymphatic tissue (MALT) and manifestation in the right lower lobe; **e** T2w overview, **f** T1w, and **g** T2w with zoom on the lesion. The ROI encloses only the solid part of the lesion; i.e., a small bronchus was spared in this case. Scale indicates 2 cm. (**c**) shows confluent areas of patchy hypointensities in a rather geographic distribution, while (**g**) shows small hypointense spots in a more repetitive pattern. (**d**) and (**h**) are concepts of a pixelwise representation of the structure of the two lesions. The images show differences between the two lesions in terms of the distribution of white, light gray, dark gray, and black pixels: in (**h**), pixels are homogeneously distributed throughout the image, while in (**d**), black and darker gray pixels are clustered in the upper right part of the image

ROIs were drawn in T1w and T2w using the freely available software “3D Slicer” (version 4.10) [24]. Every lesion was marked on every slice where it was clearly visible, resulting in a volume that was considered in the further analysis. Only solid parts were marked; i.e., blood vessels, bronchi, and the perilesional spaces were excluded. For illustration, examples of ROIs are given in Fig. 2. In case of several lesions, these were considered in the same order in T1w and T2w. The readers were blinded of any diagnostic or clinical data.

First-order statistics were extracted from the original images using HeterogeneityCAD (Commit 27ade9a) [17] in a first approach and, subsequently, PyRadiomics version 2.1.2 [18] to crosscheck the results for entropy and uniformity, after they did not show the expected inverse behavior.

The default settings for HeterogeneityCAD were left unchanged. For PyRadiomics, as recommended to make results more comparable, images were normalized and a voxel array shift was applied in the analysis. The configuration was set with the resampling adjusted to the slice thickness as follows: imageType: Original: {} \ featureClass: firstorder: \ setting: normalize: true, normalizeScale: 100, interpolator: “sitkBSpline”, resampledPixelSpacing: [2, 2, 2] for T1w or resampledPixelSpacing: [3, 3, 3] for T2w, binWidth: 5, voxelArrayShift: 300.

ROIs to calculate NICQs were placed as reported in a previous study [15]: T2NICQs were calculated from signal intensities of the lesion, muscle, and fat $\left(\left(\frac{{\mathrm{SI}}_{\mathrm{Lesion}}-{\mathrm{SI}}_{\mathrm{Muscle}}}{{\mathrm{SI}}_{\mathrm{Fat}}-{\mathrm{SI}}_{\mathrm{Muscle}}}\right)\ast 100\right)$, T1Qmean from signal intensities of the lesion and muscle $\left(\frac{{\mathrm{SI}}_{\mathrm{Lesion}}}{{\mathrm{SI}}_{\mathrm{Muscle}}}\right)$; for T2NICQ90th the 90^th^ percentile of the signal intensity of the lesion, for all other measurements, the mean signal intensity was used.

### Statistical analysis

Statistical tests were performed on the results of the first reading by S.N.. In case of multiple lesions in a patient, the largest lesion was defined as the leading lesion. Categorial parameters are given as frequencies. All metric data were tested for normal distribution using the Shapiro-Wilk test. For normally distributed data, descriptive statistics are given as mean and standard deviation. If no normal distribution was found, median and interquartile range are given. The Mann-Whitney *U* (MWU) test was used to test for significant differences of the parameters and ROC analysis was used to determine the diagnostic performance based on the leading lesions and on all lesions. For lesion-based ROC testing, an additional adjustment for clustering according to Obuchowski was done [25]. Resulting AUCs were rated (70–80% acceptable, 80–90% excellent, 90–100% outstanding) [26] and compared with those of the NICQs using the DeLong test [27].

For assessment of interrater agreement, ICC estimates and their 95% confidence intervals were calculated based on a mean-rating (*k* = 2), absolute-agreement, 2-way random effects model. For assessment of intrarater agreement, ICC estimates and their 95% confidence intervals were calculated based on a mean-rating (*k* = 2), absolute-agreement, 2-way mixed effects model. Intra- and interrater reliability was rated (ICC < 0.5 poor, 0.5–0.75 moderate, 0.75–0.9 good, > 0.9 excellent) [28].

Statistical analysis was performed using SPSS (SPSS Statistics, version 25.0, IBM Corp.) and R (version 3.5.1). For all tests, a *p* value < 0.05 was considered statistically significant.

## Results

### Study group

Sixteen patients with fungal infections and 19 patients with pulmonary lymphoma manifestations were included into the analysis. Further details are provided in Table 1. The mean scan duration was 5:24 min (range 2:48–8:08 min).

**Table 1:** Patient demographics and data of the included lesions. Mean values and standard deviation are provided for age; median and interquartile range for lesion volumes

	Lymphoma	Fungal infection
Age	54.8 ± 12.4 years	52.4 ± 18.3 years
Sex distribution (female:male)	7:12	6:10
*n* (patient/lesion)	19/38	16/33
Primary disease	1 acute myeloid leukemia, 1 acute lymphocytic leukemia, 1 chronic lymphocytic leukemia, 10 B cell non-Hodgkin lymphoma, 1 T cell non-Hodgkin lymphoma, 5 Hodgkin lymphoma	11 acute myeloid leukemia, 3 acute lymphocytic leukemia, 1 Hodgkin lymphoma, 1 severe anaplastic anemia
Pathogen in patients	-	6 *Aspergillus fumigatus*
		1 *Candida albicans*
		1 *Candida dubliniensis*
		8 no definite identification
Volume of leading lesion	Median 2020 mm^3^ [291–4414 mm^3^]	Median 208 mm^3^ [103–1176 mm^3^]
Combined volume of all lesions evaluated	Median 2269 mm^3^ [875–7690 mm^3^]	Median 352 mm^3^ [173–1442 mm^3^]
Reference standard	10 histopathology	8 microbiology
	9 clinical response to treatment	8 clinical response to treatment

### Texture analysis parameters

Data were continuous, but not normally distributed according to Shapiro-Wilk tests. Table 2 summarizes the analysis for the leading lesion and Table 3 for all lesions including adjustment for clustered data.

**Table 2:** Patient-based ROC analysis of the leading lesions. Results based on analysis of 16 fungal lesions and 19 lymphoma manifestations and results of PyRadiomics. Additionally, the median and IQR of HeterogeneityCAD results are listed. Cutoff value and direction are only specified in case of significant differences. *IQR*, interquartile range; *MWU*, Mann-Whitney *U*; *NICQ*, non-enhanced imaging characterization quotient

	Parameter	Entity	PyRadiomics													HeterogeneityCAD	
			Median	IQR	AUC [%]	AUC 95%-CI [%]	MWU *p*	Sensitivity [%]	Specificity [%]	PPV [%]	NPV [%]	Accuracy [%]	Youden’s index	Cutoff	Values indicating lymphoma in relation to cutoff	Median	IQR
T1w	Skewness	Fungal	− 0.13	− 0.42 to 0.07	58.5	42–75	0.40	47.4	81.2	75.0	56.5	62.9	0.29	n. a.	n. a.	0.08	− 0.32 to 0.26
		Lymphoma	− 0.03	0.22–0.26												0.04	− 0.17 to 0.30
	Kurtosis	Fungal	2.63	2.1–3.14	58.9	43–75	0.38	100.0	31.2	63.3	100.0	68.6	0.31	n. a.	n. a.	− 0.56	− 0.88 to − 0.04
		Lymphoma	2.84	2.50–2.97												− 0.06	− 0.32 to 0.25
	Entropy	Fungal	3.52	3.08–4.04	80.2	67–93	< 0.005	63.2	87.5	85.7	66.7	74.3	0.51	4.16	≥	46.74	10.75–466.87
		Lymphoma	4.22	3.81–4.48												1015.14	57.77–4616.74
	Uniformity	Fungal	0.10	0.07–0.13	79.6	66–93	< 0.005	57.9	93.8	91.7	65.2	74.3	0.52	0.06	≤	113.00	35.0–1190.00
		Lymphoma	0.06	0.05–0.08												2533.00	133.00–26,797.00
	Energy	Fungal	5,493,452.02	1,589,272.96–16,895,366.49	77.0	63–91	< 0.01	68.4	81.2	81.2	68.4	74.3	0.50	36,098,434.08	≥	1,191,503	338,880–5,058,725
		Lymphoma	46,742,671.37	10,869,178.24–81,239,302.99												20,572,531	2,118,027–44,187,869
	T1Qmean	Fungal	0.82	0.68–0.92	60.2	44–76	0.32	73.7	56.2	66.7	64.3	65.7	0.30	n. a.	n. a.	0.82	0.68–0.92
		Lymphoma	0.88	0.82–0.92												0.88	0.82–0.92
T2w	Skewness	Fungal	0.05	− 0.13 to 0.61	54.9	39–71	0.64	57.9	68.8	68.8	57.9	62.9	0.27	n. a.	n. a.	0.02	− 0.13 to 0.39
		Lymphoma	0.50	− 0.16 to 0.67												0.58	0.02–0.84
	Kurtosis	Fungal	2.20	1.84–3.55	64.8	49–81	0.15	78.9	62.5	71.4	71.4	71.4	0.41	n. a.	n. a.	− 0.32	− 0.93 to 0.09
		Lymphoma	2.65	2.38–3.69												− 0.13	− 0.47 to 0.63
	Entropy	Fungal	2.30	2.10–2.59	54.3	38–71	0.69	47.4	75.0	69.2	54.5	60.0	0.22	n. a.	n. a.	26.75	5.00–426.99
		Lymphoma	2.48	2.05–2.98												1862.55	509.92–4838.30
	Uniformity	Fungal	0.22	0.18–0.25	52.0	36–68	0.86	42.1	75.0	66.7	52.2	57.1	0.17	n. a.	n. a.	87.00	35.00–957.00
		Lymphoma	0.22	0.15–0.29												6204.00	1127.00–19,005.00
	Energy	Fungal	1,456,005.39	778,937.83–3,583,723.51	79.9	67–93	< 0.005	84.2	81.2	84.2	81.2	82.9	0.65	4,477,406.27	≥	5,311,273	2,073,693–44,863,860
		Lymphoma	11,725,721.57	7,479,628.68–21,624,905.53												24,790,691	11,897,372–109,549,995
	T2NICQmean	Fungal	23.52	4.82–41.43	72.0	57–87	< 0.05	84.2	68.8	76.2	78.6	77.1	0.53	19.01	≤	23.52	4.82–41.43
		Lymphoma	4.09	− 0.56 to 14.23												4.09	− 0.56 to 14.23
	T2NICQ90th	Fungal	51.55	13.85–65.04	70.4	55–85	< 0.05	100.0	50.0	70.4	100.0	77.1	0.50	50.56	≤	51.55	13.85–65.04
		Lymphoma	20.13	7.07–34.24												20.13	7.07–34.24

**Table 3:** Non-clustered ROC and clustered ROC analysis of all lesions. Results based on analysis of 33 fungal lesions and 38 lymphoma manifestations and results of PyRadiomics. Additionally, the median and IQR of HeterogeneityCAD results are listed. Cutoff value and direction are only specified in case of significant differences. *IQR*, interquartile range; *MWU*, Mann-Whitney *U*; *NICQ*, non-enhanced imaging characterization quotient

	Parameter	Entity	PyRadiomics															HeterogeneityCAD	
			Median	IQR	AUC [%]	AUC 95%-CI [%]	MWU *p*	Sensitivity [%]	Specificity [%]	PPV [%]	NPV [%]	Accuracy [%]	Youden’s index	Cutoff	Values indicating lymphoma in relation to cutoff	Clustered AUC [%]	Clustered AUC 95% [%]	Median	IQR
T1w	Skewness	Fungal	− 0.06	− 0.40 to 0.13	53.0	41–65	0.67	39.5	75.8	65.2	52.1	56.3	0.15	n. a.	n. a.	53.0	41–65	0.05	− 0.23 to 0.30
		Lymphoma	− 0.03	− 0.33 to 0.20														0.03	− 0.19 to 0.30
	Kurtosis	Fungal	2.71	2.20–3.29	53.4	42–65	0.63	100.0	24.2	60.3	100.0	64.8	0.24	n. a.	n. a.	53.4	40–66	− 0.45	− 0.80 to 0.05
		Lymphoma	2.88	2.59–3.06														− 0.05	− 0.37 to 0.25
	Entropy	Fungal	3.78	3.36–4.16	73.0	63–83	< 0.001	71.1	66.7	71.1	66.7	69.0	0.38	4.02	≥	73.1	59–87	87.28	14.75–650.79
		Lymphoma	4.18	3.93–4.51														2353.17	337.53–10,393.15
	Uniformity	Fungal	0.08	0.07–0.11	73.1	63–83	< 0.001	60.5	75.8	74.2	62.5	67.6	0.36	0.07	≤	73.1	60–86	224.00	54.50–2063.50
		Lymphoma	0.06	0.05–0.07														8439.00	752.00–72,185.00
	Energy	Fungal	6,602,482.72	3,027,347.50–32,464,709.20	77.8	68–88	< 0.0001	81.6	69.7	75.6	76.7	76.1	0.51	11,039,725.46	≥	77.8	65–90	2,871,630	638,011–8,310,936
		Lymphoma	53,556,250.85	18,054,695.78–131,990,284.17														20,675,200	5,975,740–63,000,615
	T1Qmean	Fungal	0.83	0.70–0.89	65.0	54–76	< 0.05	63.2	66.7	68.6	61.1	64.8	0.30	0.85	≥	65.0	48–82	0.83	0.70–0.89
		Lymphoma	0.88	0.82–0.97														0.88	0.82–0.97
T2w	Skewness	Fungal	0.00	− 0.34 to 0.54	63.0	52–74	0.06	60.5	72.7	71.9	61.5	66.2	0.33	n. a.	n. a.	63.0	49–77	0.08	− 0.19 to 0.44
		Lymphoma	0.41	− 0.14 to 0.69														0.49	0.10–0.83
	Kurtosis	Fungal	2.24	2.10–3.23	63.1	52–74	0.06	76.3	57.6	67.4	67.9	67.6	0.34	n. a.	n. a.	63.1	50–76	− 0.36	− 0.82 to 0.02
		Lymphoma	2.65	2.34–3.70														− 0.12	− 0.63 to 0.35
	Entropy	Fungal	2.51	2.19–3.24	54.5	43–66	0.53	15.8	100.0	100.0	50.8	54.9	0.16	n. a.	n. a.	54.5	37–71	91.65	11.38–526.76
		Lymphoma	2.72	2.08–3.59														1931.14	519.84–5176.96
	Uniformity	Fungal	0.20	0.12–0.25	52.8	41–64	0.69	15.8	100.0	100.0	50.8	54.9	0.16	n. a.	n. a.	52.9	36–70	226.00	42.00–1074.00
		Lymphoma	0.18	0.10–0.28														5675.00	1202.00–19,005.00
	Energy	Fungal	2,023,677.45	1,109,055.76–6,915,745.33	79.7	70–89	< 0.0001	76.3	78.8	80.6	74.3	77.5	0.55	7,281,428.07	≥	79.7	69–91	7,783,182	1,928,752–32,740,080
		Lymphoma	13,815,814.06	7,344,433.37–28,408,097.66														75,864,984	12,207,449–135,023,060
	T2NICQmean	Fungal	20.61	8.79–35.21	64.5	53–76	< 0.05	86.8	42.4	63.5	73.7	66.2	0.29	23.99	≤	64.5	48–81	20.61	8.79–35.21
		Lymphoma	11.07	1.47–21.70														11.07	1.42–22.01
	T2NICQ90th	Fungal	37.35	20.28–64.38	61.6	50–73	0.09	92.1	39.4	63.6	81.2	67.6	0.32	n. a.	n. a.	61.6	46–77	37.35	20.28–64.38
		Lymphoma	28.86	9.38–45.67														28.86	9.26–46.15

The following statistical analysis was performed on the results of the PyRadiomics evaluation only, since they showed the expected inverse behavior for entropy and uniformity, contrary to the results obtained with HeterogeneityCAD. Still, the results for HeterogeneityCAD are presented to illustrate how the results by different algorithms can diverge.

For the leading lesion, the MWU test showed significant differences between fungal infiltrates and pulmonary lymphoma manifestations for T1w uniformity, energy, and entropy, as well as for T2w energy.

For the leading lesion, T1w entropy showed the best diagnostic performance, followed by T2w energy, T1w uniformity, and T1w energy with only slightly inferior results. Considering all lesions, the overall performance was slightly inferior with T2w energy showing the best performance, followed by T1w energy, T1w entropy, and T1w uniformity.

The AUCs of T1w uniformity, T1w energy, and T1w entropy as well as of T2w energy furthermore exceeded those of NICQs. For the leading lesion, the DeLong test showed a significantly different AUC only for T1w entropy vs. T1Qmean (*p* < 0.05), with other differences being only close to significance, i.e., T1w energy vs. T1Qmean (*p* = 0.09), T2w energy vs. T1Qmean (*p* = 0.06), T2w energy vs. T2NICQmean (*p* = 0.07), and T2w entropy vs. T2NICQ90th (*p* = 0.06). Considering all lesions, significantly different AUCs were observed for T1w and T2w energy vs. T2NICQ90th and for T2w energy vs. T1Qmean (*p* < 0.05 each) with other differences being only close to significance, i.e., T1w energy vs. T1Qmean (*p* = 0.07) and T2w energy vs. T2NICQmean (*p* = 0.06).

### Intra- and interrater reliability

For T1w, intrarater reliability and interrater reliability were good to excellent for entropy and uniformity (ICC ≥ 0.86; *p* < 0.001). Except for the moderate intrarater agreement for energy, which was lowest of all with ICC = 0.64 (*p* = 0.05), and for kurtosis with ICC = 0.73 (*p* < 0.05), the remaining parameters in T1w also showed almost consistently good to excellent agreement (ICC ≥ 0.75).

For T2w, again entropy and uniformity showed the best intra- and interrater agreement (ICC ≥ 0.81; *p* < 0.01), but values were lower than for T1w except for a slightly higher ICC for entropy (ICC = 0.95; *p* < 0.001). All other parameters in T2w showed good-to-excellent intra- and interrater agreement (ICC ≥ 0.76; *p* < 0.05). Details are shown in Table 4.

**Table 4:** Intraclass correlation coefficient testing for intra- and interrater reliability of skewness, kurtosis, entropy, and uniformity for T1w and T2w. Results of interrater testing based on analysis of 33 fungal lesions and 38 lymphoma manifestations; results of intrarater testing based on analysis of 9 fungal lesions and 9 lymphoma manifestations. For both intrarater reliability and interrater reliability testing, results of PyRadiomics were considered. *ICC*, intraclass correlation coefficient

		Intrarater			Interrater		
	Parameter	ICC	95% CI	*p*	ICC	95% CI	*p*
T1w	Skewness	0.75	0.17–0.93	< 0.005	0.80	0.67–0.87	< 0.001
	Kurtosis	0.73	0.06–0.92	< 0.05	0.79	0.66–0.87	< 0.001
	Entropy	0.94	0.79–0.98	< 0.001	0.93	0.89–0.96	< 0.001
	Uniformity	0.94	0.79–0.98	< 0.001	0.86	0.77–0.91	< 0.001
	Energy	0.64	− 0.18 to 0.89	0.05	0.99	0.99–1.00	< 0.001
T2w	Skewness	0.90	0.66–0.97	< 0.001	0.84	0.75–0.90	< 0.001
	Kurtosis	0.76	0.14–0.93	< 0.05	0.68	0.45–0.80	< 0.001
	Entropy	0.92	0.74–0.98	< 0.001	0.95	0.92–0.97	< 0.001
	Uniformity	0.81	0.38–0.95	< 0.01	0.82	0.72–0.89	< 0.001
	Energy	0.88	0.57–0.96	0.001	0.97	0.96–0.98	< 0.001

## Discussion

This study shows that first-order statistics of texture analysis from 3-T MR images provides good overall diagnostic accuracy and useful supplementary information to enhance the differentiation of fungal infiltrates and pulmonary lymphoma manifestations in hematological patients.

The imaging data can be acquired with a speed-optimized MRI protocol including fast T1- and T2-weighted sequences that have been shown to be suitable for immunocompromised patients and have also been used to evaluate NICQs [15, 23]. The present results show that first-order statistics can improve the diagnostic performance of nonenhanced pulmonary MRI while maintaining a short examination time. It is relevant to keep the examination time as short as possible, since this group of patients unlikely tolerates prolonged MRI examination times [1]. Reliable classification and knowledge of the underlying entity of pulmonary lesions is essential, as it can spare patients invasive procedures and allows earlier initiation of appropriate treatment, such as antifungal therapy opposed to antineoplastic treatment in patients with pulmonary lymphoma manifestation [29].

In our results based on the analysis of PyRadiomics, T1w uniformity, entropy, and energy along with T2w energy showed the best performances for differentiating pulmonary lymphoma and fungal pneumonia.

Uniformity is a measure of the homogeneity with greater values implying a smaller range of discrete signal intensity values. Entropy specifies the uncertainty/randomness in the image values and measures the average amount of information required to encode the image values. Energy is a measure of the magnitude of the histograms’ voxel values and correlates with the variation on the brightness levels [30].

When considering the leading lesion, the diagnostic performance of T1w entropy was rated excellent and was also significantly superior as compared with T1Qmean. However, not all comparisons yielded significant differences, but the performance of T1w entropy, uniformity, and energy along with T2w energy was generally higher than that of NICQs. T1w und T2w energy performed similarly when considering all or just the leading lesion, while T1w entropy and T1w uniformity showed better results when considering the leading lesion. It is also noteworthy that of the parameters with the best diagnostic performance, three were a T1w- contrary to one T2w-parameter.

A brief insight into the pathology would suggest lymphoma manifestations to present with higher uniformity and lower entropy than fungal infiltrates: Fungal infections in neutropenic patients are most commonly caused by invasive *Aspergillus* and *Candida* species [31]. Histologically, these invasive infections tend to show a suppurative inflammatory response with intra-alveolar inflammatory fluid and a granulomatous inflammatory response, occasionally surrounded by fibrosis [32]. Likewise, hemorrhage and necrosis are observed in invasive fungal lung infections [32, 33]. Cavitation is another finding in patients with pulmonary fungal infection [33].

In contrast, both primary and secondary lymphoma manifestations are histologically characterized by extra-alveolar interstitial infiltrates containing mainly densely packed mass-forming, neoplastic lymphoid cells [34, 35]. Thus, unlike inflammatory lesions, pulmonary lymphomas form homogeneous masses.

However, uniformity was lower and entropy was higher in lymphoma manifestations. It should be kept in mind that MR images are a macroscopic representation of the examined tissue and do not necessarily allow conclusions about the exact histologic tissue composition. Regarding entropy, our results are in line with those of a study by Suo et al, in which the heterogeneity of malignant and inflammatory pulmonary lesions in CT images was assessed. Though neither pulmonary lymphomas nor fungal pneumonias were included, the absolute entropy in each region was also larger in the cancer than in the inflammatory lesion group. However, this difference was not significant (all *p* > 0.05) [36].

The comparison of the lesion size was not the aim of the study, but there are obvious differences with larger volumes in the lymphoma group. However, since readers were not required to mark the lesion as a whole, sizes were not considered in the analysis. Nevertheless, size differences themselves might contribute to differentiating between fungal infections and pulmonary lymphoma manifestations. Moreover, size differences of the lesions have to be considered in the evaluation of the energy of the lesions: This parameter is volume-confounded [30]; thus, a difference in size can already lead to a difference in energy. This may also explain the low intrarater agreement of energy:

If the ROIs are not equally sized, variability is possible by the raters or by volume-confounding. Thus, energy would generally have to be considered as an unreliable parameter as soon as the ROI sizes are variable.

A further interesting point when looking at the results is that entropy and uniformity provide a means of an intrinsic quality assurance: These parameters should behave inversely [37]; i.e., increasing entropy should be associated with decreasing uniformity and vice versa. This was also the reason for us to finally base the analysis on the results of PyRadiomics. But independently of this, the results of the two algorithms also diverge in the other parameters. Since numerous parameters can be set for the analysis (e.g., resampling, normalization, or bin size to name a few), the results will also easily deviate. The congruency and behavior of individual parameters may then remain understandable, but the effects on the analysis as a whole will become more complex. This will likely still be the case, although the source code can be viewed by any user: It must be assumed that most clinicians and clinical investigators will not be familiar with the underlying algorithms. Thus, texture analysis and radiomics in a broader sense likely remain a “black box” for most users which could produce results separating entities without the investigators fully understanding. This also means that any inconclusive results may easily be adopted. Nevertheless, radiomics is considered as an emerging new means for image evaluation and a powerful tool expanding human capabilities.

This study has following limitations:

First, texture analysis was confined only to solid portions of the target lesions. Further studies should focus on analyzing different regions of a lesion as in the study of Suo et al, who analyzed and compared edge and core regions [36]. Such a study design could also provide further insights into the perilesional space of fungal nodules and the halo sign known from CT, even when it is not visible to the naked eye on MR images. Second, histopathologic proof of lymphoma or proof of the pathogen causing fungal infection was not available in all patients; therefore, the clinical response to treatment had to be used as a standard of reference in such cases.

In conclusion, T1w entropy, uniformity, and energy and T2w energy showed the best performances for differentiating pulmonary lymphoma from fungal pneumonia and outperformed NICQs. Results of the texture analysis should be checked for their intrinsic consistency to identify possible incongruities of single parameters.

## Abbreviations

EORTC/MSG: European Organization for Research and Treatment of Cancer/Invasive Fungal Infections Cooperative Group and the National Institute of Allergy and Infectious Diseases Mycoses Study Group
ICC: Intraclass correlation coefficient
MWU: Mann-Whitney *U*
NICQs: Nonenhanced imaging characterization quotients
T1w: T1-weighted
T2w: T2-weighted
